# An Eye Protector for Use with the Monocular Microscope

**Published:** 1882-07

**Authors:** 


### AN EYE PROTECTOR FOR USE WITH THE MONOCULAR MICROSCOPE.

Dr. L. Brewer Hall, of Philadelphia, has devised a simple and ingenious "eye protector" for those who work with the monocular microscope. After recounting the objections to other devices for this purpose, he describes his own as follows (*Med. and Surg. Rep.*) :

The form that I now propose consists of a small, opaque disk near the eye, supported by a wire extending from its *outer* edge downward, to a point on the tube low enough to be out of the way of the nose, then bent upward, parallel to the tube, but not touching it, and attached to a ring near the top.

I made mine of a piece of brass wire, No. 18, about 45 centimeters long ; a loop at one end, 4 centimeters in diameter, covered with a piece of black paper folded over and gummed down, forms the disk. At the other end, I made a ring to fit the draw tube, and then bent the intermediate wire. I attach mine below the flange, on the draw tube, where there is no lacquer to be scratched, but if it should be thought desirable to attach it above the flange, then the ring ought to be covered with chamois, so as not to wear the polish.

The advantages of this form are, the small size of the disk and its support, interfering with the working of the instrument and view of the stage as little as possible. The support is not in the way of the nose ; the support is elastic, not uncomfortable when touched by the nose, and striking it does not displace the stand ; it can be rotated about the tube and used with either eye alternately ; it can be easily adjusted to the eye distance of any worker ; and, lastly, it is of so simple a construction that any one can make it for himself, at a very small cost, two or three cents only, in addition to the time.

